# Aggrecan and polymeric immunoglobulin receptor in extracellular vesicles of patients with seropositive rheumatoid arthritis

**DOI:** 10.1016/j.jtauto.2026.100387

**Published:** 2026-07-08

**Authors:** Susana Castaño-López, Tulio J. Lopera, Valentina Restrepo, Luisa F. Carbal, Julieta M. Ramírez-Mejía, Andrés Hernández, Diana Gil, Juan-Camilo Díaz, Marcela Manrique-Moreno, Lara Barazzuol, Rafael Posada-Duque, Mauricio Rojas, Gloria Vásquez, Lilu Corrales-Garcia, Julián D. Arias-Londoño, Justina C. Wolters, Diana Castaño

**Affiliations:** a Grupo de Inmunología Celular e Inmunogenética, Instituto de Investigaciones Médicas, Facultad de Medicina, Universidad de Antioquia, Medellín, Colombia; b Grupo Biología del Cáncer, Instituto Nacional de Cancerología, Bogotá, Colombia; c IPS Artmedica, Medellín, Colombia; d Instituto de Química, Facultad de Ciencias Exactas y Naturales, Universidad de Antioquia, Medellin, Colombia; e Department of Biomedical Sciences, University Medical Center Groningen, University of Groningen, Groningen, the Netherlands; f Department of Radiation Oncology, University Medical Center Groningen, University of Groningen, Groningen, the Netherlands; g Grupo de Neurociencias de Antioquia, Sede de Investigación Universitaria, Universidad de Antioquia, Medellín, Colombia; h Instituto de Biología, Facultad de Ciencias Exactas y Naturales, Universidad de Antioquia, Medellín, Colombia; i Unidad de Citometría de Flujo, Sede de Investigación Universitaria, Universidad de Antioquia, Medellín, Colombia; j Grupo Programa de Estudio y Control de Enfermedades Tropicales (PECET), Sede de Investigación Universitaria, Universidad de Antioquia, Medellín, Colombia; k Departamento de Ingeniería de Sistemas y Ciencias Computacionales, Universidad de Antioquia, Medellín, Colombia; l Department of Pediatrics, University Medical Center Groningen, University of Groningen, the Netherlands; m Interfaculty Mass Spectrometry Center, University Medical Center Groningen, University of Groningen, the Netherlands; n Institute for Immunology and Immune Health, Perelman School of Medicine, University of Pennsylvania, Philadelphia, PA, USA; o Department of Microbiology, Perelman School of Medicine, University of Pennsylvania, Philadelphia, PA, USA

**Keywords:** Aggrecan, Extracellular vesicles, Proteomics, Polymeric immunoglobulin receptor, Rheumatoid arthritis, Seropositivity

## Abstract

**Background:**

The presence or absence of autoantibodies distinguishes patients with rheumatoid arthritis (RA) as seropositive (SP) or seronegative (SN), with SP generally associated with poorer prognosis. Extracellular vesicles (EVs) are important mediators of intercellular communication and may contribute to RA pathogenesis by disseminating immunologically active molecules that promote systemic inflammation. However, the protein cargo of circulating EVs associated with RA seropositivity remains poorly characterized. Therefore, we aimed to identify EV-associated proteins linked to RA and seropositivity and evaluate their relationship with clinical features.

**Methods:**

Blood-derived EVs were isolated from patients with SP and SN disease and from healthy donors (HD) and were characterized according to international guidelines. EV protein cargo was analyzed by liquid chromatography–tandem mass spectrometry (LC-MS/MS). Differentially enriched proteins were identified in patients, and their associations with serum autoantibody levels, circulating cytokines, and disease activity were assessed.

**Results:**

A total of 358 proteins were identified in EVs, most of which have been previously reported in these structures. Several complement proteins, collagens, and immunoglobulins were lower in patients with RA than in HD. Notably, aggrecan (ACAN) and the polymeric immunoglobulin receptor (PIGR), not previously described in RA-derived EVs, were specifically enriched in SP patients compared with both patients with SN RA and HD. Their enrichment was significantly associated with autoantibody levels and disease activity.

**Conclusion:**

Our findings identify ACAN and PIGR as novel EV-associated proteins linked to RA seropositivity. Their association with autoantibody levels and disease activity suggests that circulating EVs may reflect biological pathways connecting cartilage remodeling, mucosal immunity, and systemic autoimmunity, supporting their potential utility as biomarkers and contributors to disease pathogenesis.

## Highlights

• Circulating EVs from patients with rheumatoid arthritis (RA) exhibit reduced protein abundance.
• EVs from RA patients with seropositive (SP) disease had a differential protein profile.
• ACAN and PIGR proteins are specifically enriched in circulating EVs of patients with SP RA.
• ACAN and PIGR in the EV cargo may contribute to the systemic manifestations of SP RA.
• ACAN and PIGR should be explored in the future as potential biomarkers for RA.

**Table:** List of abbreviations

ACC	Accuracy
ACPA	Anti-Citrullinated Peptide Antibody
ACR	American College of Rheumatology
BCA	Bicinchoninic Acid
CCP	Cyclic-Citrullinated Peptide
CDAI	Clinical Disease Activity Index
CI	Confidence Interval
CRP	C-Reactive Protein
DAMPs	Damage-Associated Molecular Patterns
DAS28	Disease Activity Score 28
DIA	Data-Independent Analysis
DLS	Dynamic Light Scattering
DPBS	Dulbecco's Phosphate-Buffered Saline
ELISA	Enzyme-Linked Immunosorbent Assay
EULAR	European League Against Rheumatism
EVdp	Extracellular Vesicle-Depleted Plasma
EVs	Extracellular Vesicles
FC	Fold Change
FT-IR	Fourier-Transform Infrared Spectroscopy
GO	Gene Ontology
HD	Healthy Donor
ICs	Immune Complexes
IL	Interleukin
IQR	Interquartile Range
ISEV	International Society for Extracellular Vesicles
IU	International Units
LC	Liquid Chromatography
LOO-CV	Leave-One-Out Cross-Validation
MHC	Major Histocompatibility Complex
MS	Mass Spectrometry
NTA	Nanoparticle Tracking Analysis
ORs	Odds Ratios
PFP	Platelet-Free Plasma
PLS-DA	Partial Least Squares Discriminant Analysis
PPP	Platelet-Poor Plasma
PRP	Platelet-Rich Plasma
Q^2^	Predictive ability Statistic
r	Spearman's Rank Correlation Coefficient
RA	Rheumatoid Arthritis
RF	Rheumatoid Factor
RT	Room temperature
SD	Standard Deviation
SDAI	Simplified Disease Activity Index
sMC	Significant Multivariate Correlation
SN	Seronegative
SP	Seropositive
TBS-T	Tris-Buffered Saline with Tween 20
TEM	Transmission Electron Microscopy
TLR	Toll-Like Receptor
TNF	Tumor Necrosis Factor
VIP	Variable Importance in Projection

## 1 Introduction

Rheumatoid arthritis (RA) is a chronic, systemic autoimmune disease characterized by persistent joint involvement, which can lead to cartilage deterioration and bone erosion if left untreated [1]. This incurable, complex condition, whose etiology is not fully characterized, involves genetic, epigenetic, environmental, immunological, and lifestyle factors [2]. A hallmark of its immunological landscape is the loss of immune tolerance and the production of autoantibodies, most notably anti-citrullinated protein antibodies (ACPAs) and rheumatoid factor (RF). The presence or absence of these autoantibodies classifies patients as seropositive (SP) or seronegative (SN), respectively [3]. Seropositivity is a critical determinant of RA diagnosis and management and is considered a poor prognostic factor; SP individuals exhibit greater systemic inflammation, more severe joint compromise, and a higher frequency of comorbidities [4]. Furthermore, when ACPAs are present at diagnosis, they are associated with a reduced likelihood of achieving drug-free remission, thereby conferring a risk of disease chronicity [5]. However, validated biomarkers for routine clinical use in RA remain limited, underscoring the need for new approaches to improve patient prediction, stratification, and disease monitoring.

It has been proposed that the initial breach of immune tolerance and production of autoantibodies occur outside the joints [6], particularly at mucosal sites exposed to environmental triggers, which ultimately promotes the expansion of autoreactive immune cells and contributes to chronic synovial inflammation, joint destruction, and extra-articular manifestations [7,8]. We propose that exploring the proteomic content of blood-derived extracellular vesicles (EVs) is a promising avenue for identifying novel molecules linking systemic involvement to joint processes in RA. EVs have emerged as key mediators linking systemic and local processes in RA [9]. EVs are lipid bilayer-enclosed, non-replicative particles released by cells that carry a molecular cargo reflective of their cellular origin and physiological/pathological states [10]. They facilitate intercellular communication by transporting biomolecules and mediating endocrine and paracrine signaling under both physiological and pathological conditions [11]. In RA, EVs may contribute to the initiation and perpetuation of inflammation by transporting damage-associated molecular patterns (DAMPs) and autoantigens, including citrullinated proteins such as α-enolase, heat shock proteins, and fibrinogen [12,13]. These EV-associated molecules can be recognized by the immune system and promote proinflammatory responses, highlighting EVs as active participants in disease pathogenesis [[14], [15], [16]].

Proteomic analyses of EVs isolated from synovial fluid of patients with RA have identified proteins such as fibrinogen, vimentin, α-enolase, and their citrullinated forms, as well as immunoglobulins and MHC-related proteins [17]. Proteins associated with neutrophils (e.g., integrin α-M, integrin β-2, and myeloperoxidase) and fibroblasts (e.g., S100 calcium-binding proteins and type VI collagen) were enriched in RA compared to osteoarthritis and healthy controls [17].

Despite growing evidence supporting the role of EVs in RA pathogenesis, the proteomic composition of circulating EVs and their relationship with serological status and disease activity remain incompletely characterized. Given that circulating EVs mirror the systemic inflammatory state and pathological processes, their protein cargo may provide insights into disease-associated pathways that cannot be captured by analysis of synovial-derived vesicles or total serum. The objective of this study was to characterize the proteomic profile of circulating EVs from patients with RA (RA-EVs) according to serological status and disease activity, and to compare them with EVs from healthy donors (HD). Additionally, we aim to evaluate whether differentially enriched proteins in patients could be associated with selected clinical variables. We hypothesized that circulating RA-EVs provide insights into disease heterogeneity and the biological mechanisms underlying distinct clinical phenotypes; thus, distinct clinical and serological subgroups would exhibit differential EV-associated protein signatures. The differentially enriched proteins identified here could be studied further as biomarkers for RA to support disease stratification, prognosis, and ultimately the development of EV-based diagnostic or monitoring tools. These findings also provide a foundation for future precision medicine approaches in RA based on molecular profiling of circulating EVs.

## 2 Methods

### 2.1 Study design

This is a cross-sectional observational study conducted in cohorts of patients with RA and HD. Clinical evaluation, blood collection, and laboratory analyses were performed at a single time point; therefore, the results describe associations and do not allow inference of temporal or causal relationships. Owing to sample availability and platform-specific requirements, some analyses were conducted in partially overlapping subcohorts, and the corresponding sample sizes are indicated in the figure legends.

### 2.2 Study participants

Forty-two patients with RA who met the 2010 American College of Rheumatology/European League Against Rheumatism (ACR/EULAR) classification criteria were consecutively enrolled between 2022 and 2024 at the tertiary healthcare institution Artmedica S.A.S. (Medellín, Colombia). The control group comprised 18 clinically HD recruited from the general population who were similar in age and sex to the patients. All participants were ≥18 years old, voluntarily agreed to participate, and provided written informed consent. The study protocol was approved by the Ethics Committee of the School of Medicine at Universidad de Antioquia (Record 005, April 20/2021, CBI_010; Medellin, Colombia) and was conducted in accordance with the Declaration of Helsinki and Colombian regulations for minimal-risk research. Individuals who had received biological therapies within six months before sample collection, were pregnant, had active infections, or had a history of other chronic inflammatory, autoimmune, malignant, or systemic diseases were excluded from the study.

Sociodemographic variables (age and sex) and disease-related characteristics (serological status, disease activity, smoking history, bone erosions, and current treatment) were recorded at the time of enrollment and are summarized in Table 1. Serological status was assessed in all participants by measuring serum RF (IgM and IgG isotypes) and ACPAs (anti-cyclic citrullinated peptide antibodies, CCP3.1, IgA/IgG) using enzyme-linked immunosorbent assay (ELISA) kits (Table S1), following the protocol described below. RF positivity was defined as concentrations >6 U/mL, whereas anti-CCP positivity was defined as concentrations >20 U/mL. Patients were classified as SN when both RF and anti-CCP were negative, and as SP when at least one of the autoantibodies mentioned was above the established cut-off values. Smoking status was defined as current or former cigarette use. Information regarding bone erosions in any affected joint and treatment was obtained from the patients’ medical records.

**Table 1:** Clinical, demographic, and paraclinical characteristics of patients and controls in the study.

	**HD**		**SN**		**SP**		**p-value**
	(n = 18)		(n = 15)		(n = 27)		
*Clinic characteristics*							
**Sex (F/M)**	16/2		14/1		26/1		0.806
**Age (years)**	59.0	(43-68)	60.0	(40-69)	58.0	(42-68)	0.622
**RF IgM (IU)**	1.5	(0.1-6.4)	1.6	(0.6-12.2)	402.0	(11.5-4755.0)	<0.001
**RF IgG (IU)**	5.0	(1.9-8.4)	5.2	(0.8-7.9)	28.7	(5.3-428.4)	<0.001
**anti-CCP3 IgA/IgG (IU)**	5.3	(4.0-26.0)	4.5	(3.2-9.6)	595.3	(4.4-13009.4)	<0.001
**Bone erosion, n (%)**	-		3	(20%)	5	(19%)	1.000
**Smoking, n (%)**	-		5	(33%)	8	(30%)	1.000
*Disease activity markers*							
**CRP (mg/dL)**	0.1	(0.0-1.4)	0.1	(0.01-6.6)	0.4	(0.0-4.8)	0.136
**DAS28-CRP**	-		1.2	(1.0-5.6)	1.8	(1.0-5.8)	0.351
**CDAI**	-		0.0	(0.0-33.0)	0.0	(0.0-20.0)	0.768
**SDAI**	-		0.2	(0.0-33.2)	0.5	(0.0-20.4)	0.873
*Cytokine levels*							
**IL-1β (pg/mL)**	29.1	(27.7-31.5)	29.4	(18.5-32.2)	30.6	(17.3-60.7)	0.005
**TNF-α (pg/mL)**	22.4	(8.5-34.2)	25.6	(4.2-31.0)	31.0	(8.6-148.6)	<0.001
**IL-6 (pg/mL)**	19.2	(11.2-23.6)	19.2	(2.9-47.2)	23.6	(5.6-90.5)	0.010
**IL-8 (pg/mL)**	17.3	(12.2-22.4)	17.3	(7.8-31.3)	18.6	(13.0-46.0)	0.495
**IL-12p70 (pg/mL)**	15.6	(1.1-23.5)	11.6	(0.7-41.0)	23.6	(0.7-124.5)	0.003
**IL-10 (pg/mL)**	16.1	(11.6-22.8)	14.3	(1.3-24.0)	16.1	(1.3-35.6)	0.231
*Therapy*							
**Methotrexate, n (%)**	-		10	(66.7%)	22	(81.5%)	0.451
**Leflunomide, n (%)**	-		5	(33.3%)	17	(63.0%)	0.107
**Hydroxychloroquine/Chloroquine, n (%)**	-		5	(33.3%)	12	(44.4%)	0.531
**Metylprednisolone/Prednisolone, n (%)**	-		4	(26.7%)	14	(51.9%)	0.193

Values are shown as the median ± interquartile range (IQR) or n (%). *p-*values for comparisons with three groups (HD, SN, and SP) were calculated using the Kruskal–Wallis test for continuous variables and Fisher's exact test for categorical variables. Comparisons between two groups (SN and SP) for DAS28, CDAI, and SDAI were completed using the Mann–Whitney *U* test. HD: Healthy donor; SN: Seronegative; SP: Seropositive; RF: Rheumatoid factor; anti-CCP3: Anti-cyclic citrullinated peptides v3.1; IU: International units; CRP: C-reactive protein; DAS28: Disease activity score 28; CDAI: Clinical disease activity Index; SDAI: Simplified disease activity index.

Disease activity was assessed using the Disease Activity Score in 28 joints (DAS28) based on C-reactive protein (CRP). Tender and swollen joint counts (28 joints) were obtained from clinical records corresponding to the study visit, whereas serum CRP levels were measured by immunoturbidimetry in an accredited clinical laboratory. DAS28-CRP scores were subsequently calculated by using the MedCalc calculator (https://www.mdcalc.com/) to classify patients as having active disease (DAS28-CRP >2.6) or inactive disease/remission (DAS28-CRP ≤2.6) [18]. As confirmatory scores, clinical disease activity index (CDAI) and simple disease activity index (SDAI) were also evaluated. Blood sampling was performed within ±7 days of clinical assessment.

### 2.3 EV enrichment

Anticoagulated peripheral venous blood (20 mL) was collected under fasting conditions in sodium citrate tubes (0.109 M, Improve, Guangzhou, China) using non-traumatic puncture. Blood was centrifuged at 300 *×g* for 10 min at room temperature (RT, 20–25°C) to obtain platelet-rich plasma (PRP). The remaining blood was sequentially centrifuged at 500 *×g* and 1800 *×g* for 10 min at RT, with PRP collected and pooled at each step. The PRP was diluted 1:4 with Dulbecco's phosphate-buffered saline (DPBS; Table S1) and centrifuged at 500 *×g* for 10 min at RT. The supernatant was then centrifuged at 3000 *×g* for 20 min at RT to obtain platelet-poor plasma (PPP), which was subsequently filtered through a 1.1 μm acrodisc (Nalgene syringe filter, Thermo Scientific) to generate platelet-free plasma (PFP).

PFP was aliquoted (1 mL) and centrifuged at 17000 *×g* for 60 min at RT. After careful removal of 900 μL of supernatant, the pellet was either used immediately for characterization or washed with DPBS and centrifuged under the same conditions, then stored at −80°C. All procedures were performed at RT.

### 2.4 Flow cytometry

EVs recovered from 1 mL of PFP were suspended in a total of 300 μL of DPBS, and acquisition was performed on the CytoFlex instrument (Beckman Coulter, Brea, CA, USA) using CytExpert software (Beckman Coulter), with the Violet-SSC-H parameter and at a constant flow rate of 30 μL/min. DPBS alone was acquired under the same conditions to determine the electronic noise threshold. The EVs' size was estimated using commercial fluorescent beads of known sizes (Megamix-Plus SSC and FSC; 0.1, 0.16, 0.20, 0.24, 0.3, 0.5, and 0.9 μm; Table S1) and platelets (as a biological control ≥1 μm).

EVs were stained with fluorescent-labeled antibodies, as previously reported [19]. Briefly, 100 μL of freshly enriched EVs were stained using specific monoclonal anti-human antibodies targeting CD41a (platelets), CD45 (leukocytes), CD105 (endothelial cells), CD235a (erythrocytes), and CPs (citrullinated proteins), as well as F(ab’)2 fragments against human IgG and IgM (Table S1) and were incubated for 1 h at RT. Independently, the vesicles were stained with 5 μL of a 1 μg/μL FM4-64 solution (Table S1) at 37°C for 30 min. Then, all stains were washed with 1 mL of DPBS at 17,000 ×g for 60 min at RT. In all cases, stained EVs were acquired using an LSRFortessa flow cytometer (Becton Dickinson) with FACSDiva software. To confirm the vesicular nature of the enriched structures, some EV samples stained with the anti-CD235a monoclonal antibody were briefly (∼5 s) treated with 0.05% v/v Triton X-100 solution and re-acquired under the same conditions. Data analysis was performed with FlowJo™ v10.8.1 (Tree Star, Inc., USA)

### 2.5 Nanoparticle tracking analysis (NTA)

EVs were randomly selected per study group, thawed, resuspended in 1 mL of DPBS, and analyzed using the Nanosight NS300 system (Malvern Instruments, Malvern, UK). For each sample, five 60-s videos were recorded. Analysis was performed at 25 frames per second, with a detection threshold of 4 and a gain of 15–73. The software processed all five videos and reported the average size distribution and concentration per sample. Between readings, DPBS was injected to clean until no particles were detected.

### 2.6 Transmission electron microscopy (TEM)

EVs from randomly selected individuals were thawed at RT, fixed with 2.5% glutaraldehyde at 4°C, deposited onto carbon-coated copper TEM grids (Formvar/carbon 200 mesh copper grids, Sigma-Aldrich, St. Louis, MO, USA), air-dried, and contrasted with 4% uranyl acetate for 8 min at RT. The samples were examined using a Tecnai G2 F20 microscope (FEI Company, Hillsboro, OR, USA) at the "Centro de Microscopía Avanzada" of the Universidad de Antioquia. A general field scan was performed, and representative fields were photographed at specified magnifications.

### 2.7 Fourier-transform infrared (FT-IR) spectroscopy

EVs were thawed at RT and then loaded individually in a BioATR Cell integrated with a Tensor II spectrometer and a liquid nitrogen Mercury Cadmium Telluride detector (Bruker Optics, Ettlingen, Germany). Each sample was loaded with 20 μL, and the solvent (DPBS) was evaporated at 65°C. Acquisition was performed with a spectral resolution of at least 0.04 cm−1 and 120 scans per spectrum. All samples were acquired on the same day. The background was taken under the same conditions as the empty cell.

### 2.8 Autoantibody detection by ELISAS

Serum from all individuals was collected, aliquoted, and stored at −80°C until analysis. One aliquot per donor was thawed on ice, diluted 1:100, and incubated for 1 h at RT in commercially available, previously blocked, ELISA plates for the detection of RF and ACPAs, together with calibrators and positive and negative controls (Table S1). Several washes were performed to eliminate the unbound sample. The antigen-antibody reaction was detected using the corresponding HRP-conjugated anti-CCP3.1 IgG/IgA, RF IgG, or RF IgM reagent, respectively. After colorimetric development, absorbance was measured at 450 nm. The calibrator curve determined autoantibody units.

### 2.9 Cytokine quantification by cytometric bead array

A panel of six cytokines, including interleukin (IL) IL-6, IL-1β, IL-12p70, IL-10, IL-8, and TNF-α, was quantified in patients and controls using a cytometric bead array system (Table S1). One serum aliquot per donor was thawed on ice, diluted 1:4, and incubated with a mixture of cytokine-specific capture beads for 1.5 h at RT. After washing, PE-conjugated detection reagent was added and incubated for an additional 1.5 h at RT. Samples were then washed, resuspended, and acquired on a CytoFLEX flow cytometer (Beckman Coulter). Cytokine concentrations were determined from the fluorescence intensity of each population and standard curves.

### 2.10 Western blot (WB)

EVs and EV-depleted plasma (EVdp) samples were treated with RIPA buffer supplemented with protease inhibitors (1:100) (Table S1). Protein concentration was determined using the Pierce BCA assay (Table S1), and 10 μg of protein per sample was denatured in loading buffer and separated by tricine–SDS-PAGE, 4–10% or 4–7.5% gradient gels. Proteins were transferred to nitrocellulose membranes using a Trans-Blot Turbo system (Bio-Rad). Membranes were stained with Ponceau S (Table S1) to assess total protein loading. Then, blocked with 5% non-fat milk in Tris-buffered saline with Tween 20 (TBS-T), and incubated overnight at 4°C with the primary antibody (Table S1). IRDye 800-conjugated secondary antibody was used, and fluorescence was detected using the Odyssey infrared imaging system (Li-COR).

### 2.11 Liquid chromatography-tandem mass spectrometry (LC-MS/MS) proteomics and data pre-processing

Five individuals per experimental group were selected based on the highest percentages of FM4-64-positive EVs and on higher/lower autoantibody titers (SP/SN) and DAS28 representative values (active/inactive disease) for each study group. In-gel digestion was performed on 10 μg of protein per sample, using 16.7 ng/μL sequencing-grade trypsin (Table S1) after cysteine reduction with 10 mM dithiothreitol and alkylation with 55 mM iodoacetamide.

Label-free quantification was performed using an Orbitrap quadrupole mass spectrometer equipped with a nano-electrospray ion source (Orbitrap Exploris 480, Thermo Scientific). The sample was separated via liquid chromatography (LC) using an Evosep One system (Evosep Biosystems, Odense, Denmark) equipped with a nano-column (EV1137 Performance column, 15 cm × 150 μm, 1.5 μm, Evosep). The mobile phase consisted of buffer A (0.1% v/v formic acid in Milli-Q water) and buffer B (0.1% v/v formic acid in acetonitrile). The mass spectrometer was operated in positive ion mode and in data-independent acquisition (DIA), with 16 mass-to-charge (*m*/*z*) isolation windows, a precursor mass range of 400–1000 *m*/*z*, FAIMS compensation voltages of 45–60 V, and three scans per screening cycle. LC-MS/MS raw data were processed with Spectronaut (version 18.1.230626.50606, Biognosys) using the standard settings of the directDIA workflow, except that quantification was performed on MS1 and identification on MS2 with a human SwissProt database (www.uniprot.org, version UP000005640, 20586 entries). For quantification, local normalization was applied. The search included carbamidomethylation (cysteine) as a fixed modification and acetylation (N-terminal), oxidation (methionine), and deamidation (of arginine to citrulline) as variable modifications. In total, the software identified 524 proteins in the EVs. All procedures were conducted at the Interfaculty Mass Spectrometry Center of the University Medical Center Groningen (UMCG, Groningen, The Netherlands).

The proteomics data were deposited in the ProteomeXchange Consortium via the PRIDE Partner repository [20] with the dataset identifier PXD076240.

### 2.12 Proteomic data processing

All data were analyzed using Microsoft Excel and R. Proteins matched those reported by Frankenfield et al. [21] as “contaminants” arising from sample handling were removed. Using Perseus software version 2.0.10.0 (Max Planck Institute of Biochemistry, Planegg, Germany), a row-based filter was applied to retain only proteins with at least 3 valid signals per experimental group. A final list of proteins was obtained, with an overall missing rate of 2.3% [22]. The mean of the corresponding group was used to impute the remaining missing values. Vesiclepedia (Version 4.1, 2018) and Exocarta (July 2015) were downloaded to map previously reported EV proteins.

### 2.13 Bioinformatic analysis

For a total of 358 identified proteins, partial least squares discriminant analysis (PLS-DA) was performed in study groups, by pairs, and as an exploratory, supervised multivariate approach to assess whether the global EV-associated proteomic profile showed preliminary separation between study groups, following a previously described procedure [23], using code implemented in MATLAB (MathWorks, Natick, MA, USA). We applied the variable selection method “Significant Multivariate Correlation” index (sMC) to perform the analysis [24]. The top 20 proteins with the highest sMC values were selected as the most relevant for distinguishing between comparison groups. To reduce the risk of overfitting, model complexity was optimized using the leave-one-out cross-validation (LOO-CV) approach [25]. Variable importance measures were derived within the cross-validation framework. Model performance and discriminative capacity were assessed using cross-validated metrics, including accuracy (ACC) and the predictive ability statistic (Q^2^).

For the top proteins, a fold-change (FC) analysis was performed using a log2 transformation, and proteins with an FC ≥ 0.6 were considered differentially enriched proteins (DEPs).

Gene functional enrichment analysis was performed using Enrichr (https://maayanlab.cloud/Enrichr/), specifically Gene Ontology (GO) Jensen COMPARTMENTS [26], GO Biological Process 2023, GO Cellular Component 2023, and GO Molecular Function 2023 [27], using the DEP list from each comparison. Only pathways showing statistically significant enrichment (adjusted p-value <0.05) were considered for further interpretation. STRING database (https://string-db.org/) [28] was used to predict protein-protein interaction networks, using sMC-top20. The networks were graphed using Cytoscape v3.10.1.

### 2.14 Statistical analysis

Data normality was assessed using the Shapiro-Wilk test. Parametric data are presented as the mean ± standard deviation (SD) and were analyzed using one-way analysis of variance (ANOVA) followed by Bonferroni's multiple-comparison *post hoc* test. Non-parametric data are presented as median ± interquartile range (IQR) and were analyzed using the Kruskal-Wallis test followed by Dunn's multiple-comparison *post hoc* test. Comparisons between two groups were performed using the non-parametric two-tailed Mann–Whitney *U* test.

Associations of categorical variables between patients with SN and SP RA were performed using Fisher's exact test. Associations between continuous variables, including protein abundance, autoantibody levels, disease activity scores, and cytokine concentrations, were assessed using Spearman's rank correlation coefficient (r).

Odds ratios (ORs) and 95% confidence intervals (CIs) were estimated by age-adjusted logistic regression models. Protein abundance values were log2-transformed before analysis. To reduce small-sample bias and address potential complete or quasi-complete separation, associations were evaluated using Firth's penalized logistic regression.

The statistical test applied in each analysis is indicated in the corresponding figure legend. Statistical significance was defined as p ≤ 0.05, with significance levels denoted as follows: ∗p ≤ 0.05; ∗∗p ≤ 0.01; ∗∗∗p ≤ 0.001; and ∗∗∗∗p ≤ 0.0001. Only significant differences are displayed in the figures. All statistical analyses were performed in RStudio (Posit, Boston, MA, USA), and figures were generated using RStudio and/or GraphPad Prism version 9.0.0 (GraphPad Software, Boston, MA, USA).

## 3 Results

### 3.1 Demographic and clinical characteristics of patients and HD

Most participants were women (56/60 [93.5%]), and the median age of all individuals, including HD, was 59 years (IQR: 40 – 68). Only patients with SP RA had positive levels of RF (IgM: 402.0 IU; IQR: 11.5 – 4755.0 and IgG: 28.7 IU; IQR: 2.3 – 428.4) and ACPAs (anti-CCP3 IgA/IgG: 595.3 IU; IQR: 4.4 – 13009.4) and were statistically significant when compared to HD and SN (p < 0.001). No significant differences were observed between patients with SN and SP RA regarding the prevalence of smoking (33% vs. 30%; OR 0.95; 95% CI = [0.26 – 3.44]; p = 1.00) or bone erosion (20% vs. 19%; OR 0.92; 95% CI = [0.19–4.46]; p = 1.00). In this study, we included patients with clinical remission and active disease in both the SN (DAS28: 1.2 [1.0-5.6]) and SP (DAS28: 1.8 [1.0-5.8]) groups, with no statistically significant difference between them (p = 0.351). As indicators of systemic inflammation, serum levels of IL-1β, TNF-α, IL-6, and IL-12p70 were significantly higher in patients with SP RA than in HD, irrespective of disease activity (Table 1). We did not observe differences in IL-8 and IL-10 levels. Most patients received methotrexate as treatment (either in combination with other medications or alone), and the protocols were comparable across groups (p = 0.451).

### 3.2 Enriched structures mainly correspond to EVs of medium size

**Fig. 1:**
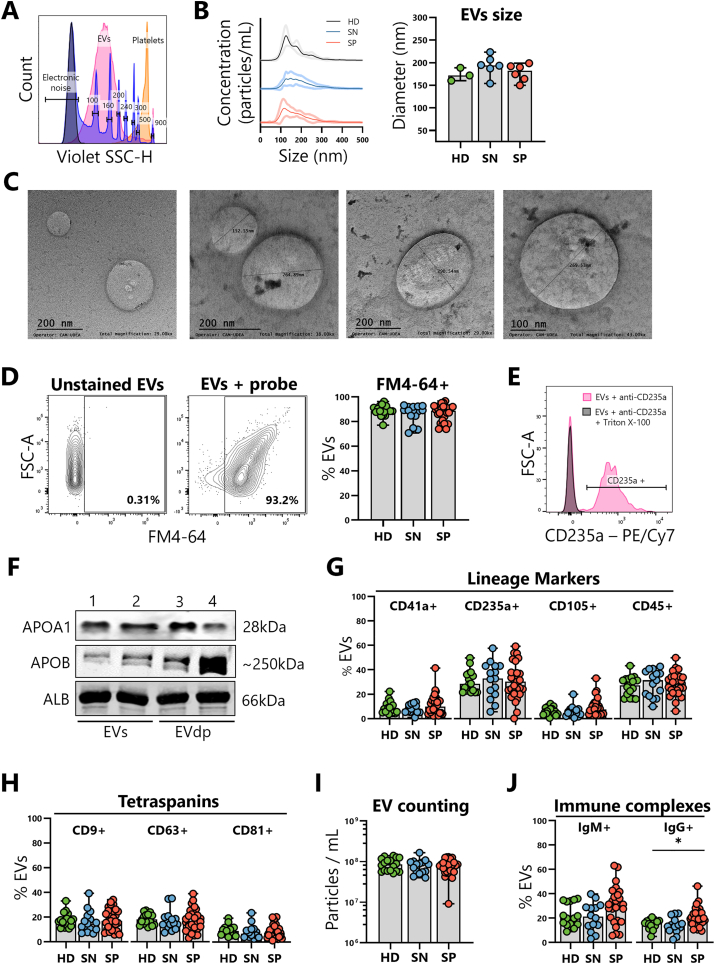
Similar morphological characteristics of EVs were observed among the study groups. **A.** Representative flow cytometry histograms showing the extracellular vesicles (EVs, pink) and platelets (orange) of a patient with seropositive (SP) rheumatoid arthritis (RA); reference beads of different sizes are shown in nanometers (nm, dark blue). Electronic noise is displayed in dark gray. **B.** Left, representing nano tracking analysis (NTA) diagrams showing the size distribution of EVs from healthy donors (HD, gray) and patients with seronegative (SN, blue) or SP (red) disease. Dark, thinner lines indicate the mean of the measurements, and light, wider lines indicate the spread of the data. Right, consolidated average size of EVs by NTA, as explained for the left side of this panel. HD (green, n = 3), SN (blue, n = 6), and SP (orange, n = 6). Data are shown as mean ± standard deviation (SD). One-way ANOVA and Bonferroni multiple comparisons. **C.** Representative micrographs of EVs from a patient with SP RA by transmission electron microscopy. The line within each EV indicates the structure's diameter. The reference size scale is indicated at the bottom left of each image. **D.** EVs labeled with the FM4-64 probe. Left, gating strategy for analyzing EVs by flow cytometry, showing the unstained sample (experimental control) to set up the region, and the stained EVs of the same donor (middle). Right, consolidated frequency of EVs that were positive for FM4-64 by flow cytometry. HD (green, n = 18), SN (blue, n = 15), and SP (orange, n = 29). Data are shown as median ± range. Kruskal-Wallis with Dunn's multiple comparisons. **E.** Representative flow cytometry histograms showing EVs stained with CD235a before (pink) and after (dark gray) treatment with Triton X-100. **F.** Representative western blots showing APOA1, APOB, and ALB levels in EVs (n = 2, lanes 1 and 2) and EVs-depleted plasma (EVdp, n = 2, lanes 3 and 4). **G-J.** Flow cytometry consolidates for: lineage markers (G); tetraspanins (H); EV counting (I); and immune complexes (J). HD (green, n = 18), SN (blue, n = 15), and SP (orange, n = 29). Kruskal-Wallis with Dunn's multiple comparisons, ∗p ≤ 0.05.

Using flow cytometry, EVs with diameters of 100-250 nm were detected in patients and HD, compared with reference beads of known sizes (Fig. 1A). The NTA showed similar EV sizes across groups, with average diameters of 173.4 ± 14.4 nm in HD, 193.2 ± 23.0 nm in SN, and 178.1 ± 18.3 nm in SP (Fig. 1B). Representative electron microscopy images confirm the EV ranges observed with previous techniques. In addition, EVs exhibited a round and intact structure that seems to be delimited by a membrane (Fig. 1C).

FM4-64 staining revealed lipidic membrane structures in the EV isolates, with approximately 88.8% of particles testing positive for this probe, and no differences were observed between study groups (Fig. 1D). Anti-CD235a positivity was rapidly abolished upon Triton X-100 treatment, supporting its association with membrane-bound components (Fig. 1E). In parallel, Western blot analysis demonstrated the presence of apolipoprotein A1 (APOA1), apolipoprotein B (APOB), and albumin (ALB) (Fig. 1F), indicating co-isolation of lipoproteins and plasma proteins, potentially as part of a biomolecular corona of EVs [29].

We further assessed the presence of transmembrane-associated molecules, including lineage markers (CD41a, CD45, CD105, and CD235a at the plasma membrane) and tetraspanins (CD9, CD63, and CD81 of endosomal origin). A high proportion of particles were positive for CD235a (30.55% ± 14.44%) and CD45 (28.57% ± 9.36%), followed by CD41a (8.80% ± 4.93) and CD105 (6.08% ± 4.62) (Fig. 1G and S1A). In addition, approximately 15-25% of particles displayed one of the three multivesicular body-associated markers (Fig. 1H and S1B). These findings support the presence of membrane-associated structures within the isolates.

The particle concentration did not differ between patients and HD, with a median of 8.32 × 10^7^ particles/mL of plasma across all groups (Fig. 1I). Regarding IC formation, a significant increase in the frequency of IgG-positive particles was observed in patients with SP RA compared with HD (SP: 20.99% ± 8.15% vs HD: 14.71% ± 4.11%, p = 0.022) (Fig. 1J and S1C).

Taken together, these findings suggest that the isolates comprise a heterogeneous population of membrane-associated particles; therefore, they are herein referred to as EV-enriched fractions, in accordance with the Minimal Information for Studies of Extracellular Vesicles (MISEV) 2023 guidelines [30].

### 3.3 Enriched EVs contain proteins previously detected in these structures

**Fig. 2:**
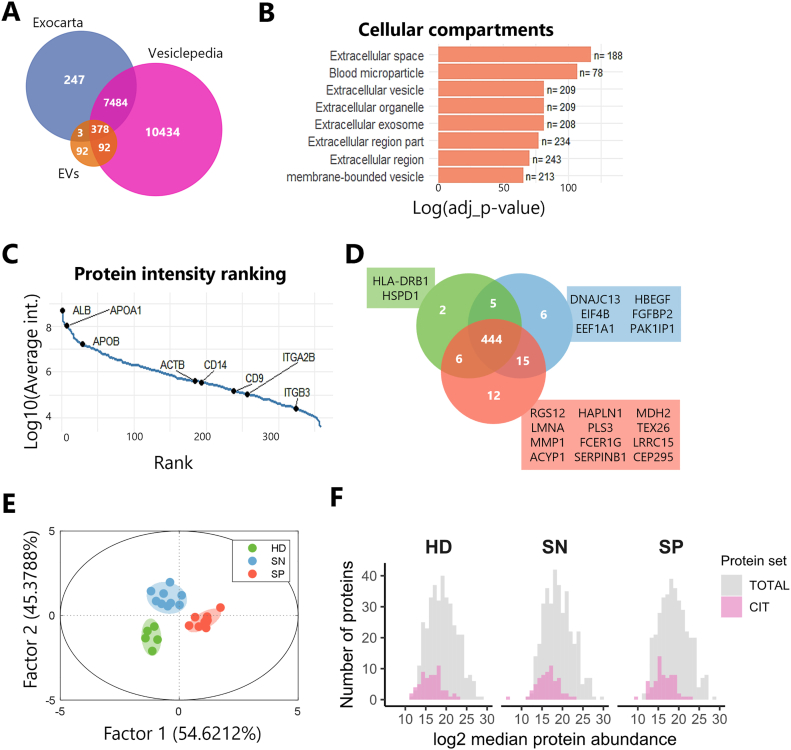
EVs from patients with RA contain proteins previously described in these structures. **A.** Venn diagram comparing proteins reported in Exocarta (dark blue) and Vesiclepedia (pink) databases with proteins detected in our EV samples (orange). **B.** Gene ontology (GO) enrichment analysis for the cellular compartment of total proteins detected in EVs from RA patients and HD. Bar graphs show the most enriched biological pathways in the library used. The terms for the input gene set are shown by -log10 of the adjusted p-value. All terms shown had p-values <0.05. The n to the right is the number of proteins that match each term. **C.** Protein intensity ranking plots for EV samples. Each blue point forming the line represents a unique protein, with the x-axis indicating its rank by intensity and the y-axis indicating the average intensity on a log10 scale. Highlighted proteins (black points) represent key EV markers (ACTB, CD14, CD9, ITGA2B, ITGB3) and abundant plasma proteins (ALB, APOA1, APOB). **D.** Venn diagram comparing the proteins detected in each of the study groups (HD, green; SN, blue; SP, orange), filtered with at least one positive signal in one donor. Adjacent rectangles highlight proteins that occur only once in their respective groups (square those). **E.** Partial least squares discriminant analysis (PLS-DA) multivariate analysis. Each factor is the percentage of the sum of variances. HD (green, n = 5), patients with RA SN (blue, n = 9), and SP (orange, n = 10). **F.** Histograms show the distribution of log2 median protein abundances. Citrullinated proteins (CIT, pink bars) are overlaid on the total detected proteome (gray bars). Histograms were generated using identical bin widths and boundaries across groups to ensure direct comparability of abundance distributions.

To confirm that the general protein composition of our EVs matches prior experimental reports, we compared our proteome with publicly available databases. According to Exocarta and Vesiclepedia, 83.7% of the proteins (473 of 565 total) identified had been previously reported in EVs by other studies [31,32] (Fig. 2A) and are significantly associated with cellular compartments corresponding to the extracellular space, blood microparticles, and EVs (Fig. 2B). The complete list of proteins detected in our samples is provided in Table S2. Additionally, the ranking of protein intensities showed that, although plasma proteins isolated as part of the EV corona (APOA1, APOB, and ALB) had high representativity, proteins associated with the EV membrane, including β-actin (ACTB), CD14, ITGA2B (also known as CD41a), and CD9, were also detected (Fig. 2C and Table S2).

Most of the proteins detected in EVs are shared across the study groups, including those associated with plasma components (Fig. 2D). Few proteins were exclusive to a single group (Fig. 2D) and had high percentages of missing values (Fig. 2D and Table S2). Therefore, we filtered the data and selected proteins with at least 3 valid values per study group, resulting in 358 proteins for further analysis (Table S3). PLS-DA reveals that the protein profiles exhibited group-specific distribution patterns. According to factor 1 (54.62%), patients with SN RA are closer to HD, whereas patients with SP RA form a separate cluster (Fig. 2E).

By screening for post-translational modifications in MS-detected proteins, citrullinated peptides were identified. The 15 proteins with the most modified peptides are summarized in Table 2. Although the global distribution of citrullinated peptides is similar among groups (Fig. 2F), proteins that have been previously associated with RA autoantigens were citrullinated in the blood EVs, such as fibrinogen and fibronectin [33]. Interestingly, in agreement with our results, another study reported that the citrullinome of RA-EVs is enriched for citrullinated complement proteins [34] (Table 2).

**Table 2:** Most frequently identified citrullinated proteins in EVs after exclusion of peptides containing only C-terminal citrullinated arginine residues.

Gene symbol	Protein name	Uniprot ID	Citrullination frequency^a^
ALB	Albumin	P02768	27
FGB	Fibrinogen beta chain	P02675	10
FGA	Fibrinogen alpha chain	P02671	9
C4A	Complement C4-A	P0C0L4	6
APOA1	Apolipoprotein A-I	P02647	4
C3	Complement C3	P01024	4
C4BPA	C4b-binding protein alpha chain	P04003	4
IGLV1-47	Immunoglobulin lambda variable 1-47	P01700	4
IGLV1-51	Immunoglobulin lambda variable 1-51	P01701	4
ITIH2	Inter-alpha-trypsin inhibitor heavy chain H2	P19823	4
C9	Complement component C9	P02748	3
CFB	Complement factor B	P00751	3
CP	Ceruloplasmin	P00450	3
FN1	Fibronectin	P02751	2
PLG	Plasminogen	P00747	2

a Citrullination frequency corresponds to the number of unique citrullinated peptides assigned to each protein after excluding peptides with citrullinated residues at the C-terminus.

These data confirm the presence of EV-derived proteins, including autoantigens, in our samples.

### 3.4 Aggrecan and polymeric immunoglobulin receptor are enriched in EVs of SP RA patients

**Fig. 3:**
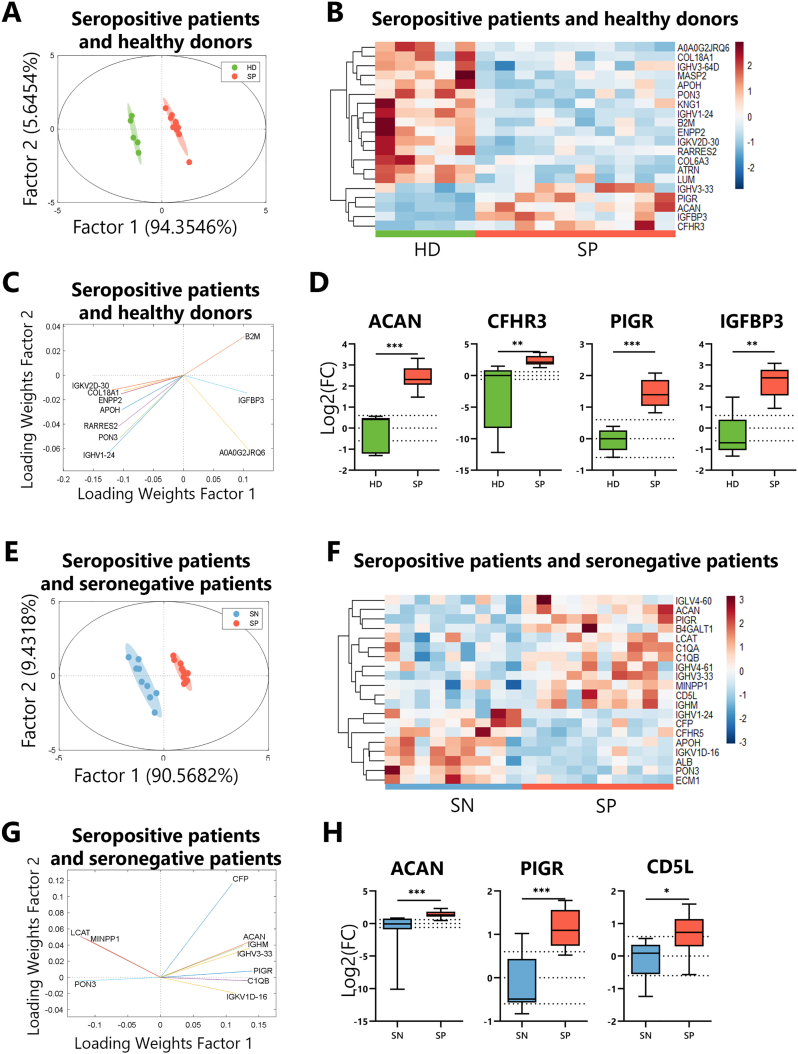
EVs from patients with SP RA exhibited a unique protein profile **A.** PLS-DA multivariate analysis. Each factor is shown as the percentage of variance. HD (green, n = 5) and seropositive RA patients (SP) (orange, n = 10). **B.** Heat map showing the abundance of the 20 proteins with the highest “Significant Multivariate Correlation” index (sMC) comparing HD and patients with SP RA, normalized by z-score using unsupervised hierarchical clustering analysis. Downregulated and upregulated proteins are shown in blue and red, respectively. **C.** PLS-DA loading plot comparing patients with SP RA and HD. The 10 proteins with the highest sMC scores are shown. Loadings on Factor 1 and Factor 2 indicate each protein's contribution to group discrimination, with positive loadings associated with patients with SP RA and negative loadings with HD. **D.** Box plots and whiskers of fold change (FC) analysis for differentially enriched proteins (DEP) that had FC > 0.6. Dotted lines denote 0 and thresholds at ±0.6. Mann-Whitney, ∗∗p ≤ 0.01; ∗∗∗p ≤ 0.001 **E.** PLS-DA multivariate analysis. Each factor is shown as the percentage of variance. Patients with SN (blue, n = 9) and SP (orange, n = 10) disease. **F.** Heat map showing the abundance of the 20 proteins with the highest sMC comparing patients with SN and SP RA, normalized by z-score using unsupervised hierarchical clustering analysis. Downregulated and upregulated proteins are shown in blue and red, respectively. **G.** PLS-DA loading plot comparing patients with RA SP and SN. The ten proteins with the highest sMC scores are shown. Loadings on Factor 1 and Factor 2 indicate each protein's contribution to group discrimination, with positive loadings associated to patients with SP RA and negative loadings to patients with SN RA. **H.** Box plots and whiskers of fold change (FC) analysis for DEP that had FC > 0.6. Dotted lines denote thresholds at ±0.6 and 0. Mann-Whitney, ∗p ≤ 0.05; ∗∗∗p ≤ 0.001.

As an initial screening approach, Fourier Transform Infrared (FT-IR) spectroscopy was used to evaluate the overall composition of EVs. However, no group-specific spectral differences were detected, indicating a similar global biochemical composition among the study groups (Fig. S2A). Next, LC-MS/MS proteomic analysis was performed to compare patients with SP disease and HD. PLS-DA revealed a clear separation between groups along the first component, which accounted for 94.35% of the model discrimination (Fig. 3A). The heatmap of the 20 proteins with the highest sMC values (Table 3) revealed distinct abundance patterns between patients with SP RA and HD (Fig. 3B). Fifteen of the 20 proteins were downregulated in patients with SP RA, and some showed significant differences compared with HD (Fig. S2B). A substantial proportion of the top-ranked proteins corresponded to immunoglobulin-related components (IGHV1-24, IGKV2D-30, A0A0G2JRQ6, IGHV3-64D, IGHV3-33). The loading plot identified the proteins that contributed most to group discrimination in the PLS-DA model (Fig. 3C). Among the proteins significantly upregulated in patients with SP RA were aggrecan core protein (ACAN; FC = 2.32, p = 0.0007), complement factor H-related protein 3 (CFHR3; FC = 2.11, p = 0.0013), polymeric immunoglobulin receptor (PIGR; FC = 1.40, p = 0.0007), and insulin-like growth factor-binding protein 3 (IGFBP3; FC = 2.39, p = 0.0023) (Fig. 3D).

**Table 3:** EV proteins with the highest significance according to sMCindex between HD and patients with SP RA.

Gene name	Protein name	sMC	VIP	Uniprot ID
IGHV1-24	*Immunoglobulin heavy variable 1-24*	54.21	2.29	A0A0C4DH33
IGKV2D-30	*Immunoglobulin kappa variable 2D-30*	34.63	2.20	A0A075B6S6
A0A0G2JRQ6	*Ig-like domain-containing protein*	21.70	1.97	A0A0G2JRQ6
RARRES2	*Retinoic acid receptor responder protein 2*	21.69	2.00	Q99969
PON3	*Serum paraoxonase/lactonase 3*	16.72	1.79	Q15166
IGFBP3	*Insulin-like growth factor-binding protein 3*	16.18	1.87	P17936
ENPP2	*Ectonucleotide pyrophosphatase/phosphodiesterase family member 2*	15.15	1.92	Q13822
APOH	*Beta-2-glycoprotein 1*	14.75	1.81	P02749
B2M	*Beta-2-microglobulin*	14.60	1.88	P61769
COL18A1	*Collagen alpha-1(XVIII) chain*	14.26	1.80	P39060
CFHR3	*Complement factor H-related protein 3*	13.53	1.75	Q02985
PIGR	*Polymeric immunoglobulin receptor*	13.43	1.96	P01833
MASP2	*Mannan-binding lectin serine protease 2*	13.10	1.84	O00187
IGHV3-64D	*Immunoglobulin heavy variable 3-64D*	12.33	1.66	A0A0J9YX35
COL6A3	*Collagen alpha-3(VI) chain*	12.00	1.69	P12111
ATRN	*Attractin*	11.81	1.72	O75882
KNG1	*Kininogen-1*	11.44	1.59	P01042
ACAN	*Aggrecan core protein*	11.07	2.01	P16112
IGHV3-33	*Immunoglobulin heavy variable 3-33*	10.67	1.55	P01772
LUM	*Lumican*	10.60	1.68	P51884

VIP: Variable importance in projection. sMC: Significat multivariate correlation

To further evaluate proteins specifically associated with seropositivity, patients with SP RA were directly compared with those with SN disease, excluding HD. PLS-DA again revealed a clear separation between groups (factor 1: 90.5682%; factor 2: 9.4318%), indicating distinct EV proteomic profiles according to serological status (Fig. 3E). Consistent with this separation, the heatmap of the top discriminant proteins showed more proteins enriched in patients with SP RA than in the previous analysis (Fig. 3F and Table 4). The corresponding loading plot identified the proteins contributing most strongly to group discrimination (Fig. 3G). Among them, ACAN (FC: 1.31, p = 0.0002) and PIGR (FC: 1.10, p = 0.0004) remained consistently increased in EVs from patients with SP disease (Fig. 3H). Additional proteins, including CD5L (FC: 0.73, p = 0.0133), C1QB (FC: 0.23, p = 0.0230), C1QA (FC: 0.16, p = 0.0453), and MINPP1 (FC: 0.42, p = 0.03299) were also significantly increased in SP compared with SN patients; however, not in every case their values exceed the predefined threshold of 0.6 FC (Fig. 3H and S2C).

**Table 4:** EV proteins with the highest significance according to sMC index between patients with SN and SP RA.

Gene name	Protein name	sMC	VIP	Uniport ID
PIGR	*Polymeric immunoglobulin receptor*	22.96	2.51	P01833
ACAN	*Aggrecan core protein*	20.68	2.53	P16112
IGHV3-33	*Immunoglobulin heavy variable 3-33*	17.11	2.46	P01772
C1QB	*Complement C1q subcomponent subunit B*	14.93	2.30	P02746
IGHM	*Immunoglobulin heavy constant mu*	13.89	2.40	P01871
PON3	*Serum paraoxonase/lactonase 3*	12.60	2.27	Q15166
LCAT	*Phosphatidylcholine-sterol acyltransferase*	11.85	2.09	P04180
CFP	*Properdin*	10.24	1.94	P27918
MINPP1	*Multiple inositol polyphosphate phosphatase 1*	9.81	1.98	Q9UNW1
IGKV1D-16	*Immunoglobulin kappa variable 1D-16*	8.84	2.00	P01601
ECM1	*Extracellular matrix protein 1*	8.51	2.22	Q16610
C1QA	*Complement C1q subcomponent subunit A*	8.48	1.89	P02745
IGHV1-24	*Immunoglobulin heavy variable 1-24*	8.43	1.83	A0A0C4DH33
CFHR5	*Complement factor H-related protein 5*	8.11	1.92	Q9BXR6
B4GALT1	*Beta-1,4-galactosyltransferase 1*	7.74	1.88	P15291
CD5L	*CD5 antigen-like*	7.65	2.06	O43866
IGHV4-61; IGHV4-59	*Immunoglobulin heavy variable 4-61/4-59*	7.11	1.81	A0A0C4DH41; P01825
ALB	*Albumin*	6.39	2.07	P02768
IGLV4-60	*Immunoglobulin lambda variable 4-60*	6.11	1.69	A0A075B6I1
APOH	*Beta-2-glycoprotein 1*	6.03	1.84	P02749

VIP: Variable importance in projection; sMC: Significat Multivariate Correlation.

Stratification of patients with SP RA by disease activity showed no significant differences in the FCs of these proteins (Fig. S3A). Because neither ACAN nor PIGR has been previously reported in circulating EVs from patients with RA, we selected ACAN for orthogonal validation in an independent cohort. Western blot analysis confirmed the presence of ACAN in EV preparations from all evaluated donors (Fig. S3B).

To assess the robustness of the PLS-DA classification models, LOO-CV analysis was performed (Table S4). In the total RA vs HD model (Fig. 2E), the exploratory model based on the top 20 proteins showed moderate discriminative performance (AUC = 0.92, Accuracy = 0.88), whereas reduction to 6 proteins (CFHR3, LGALS3BP, B2M, CFP, RARRES2, LYZ) markedly improved classification accuracy (AUC = 0.99, Accuracy = 0.96), indicating that the reduced signature captures the most relevant discriminant information (Table S4). Consistent with the marked separation observed between patients with SP RA and HD in the PLS-DA score plot (Fig. 3A), serological stratification yielded stronger, more robust models. Both the explanatory (top 20 proteins) and reduced (including the proteins RARRES2, PON3, IGFBP3, ENPP2, B2M, COL18A1, CFHR3, PIGR, MASP2, COL6A3, ACAN) SP vs HD models achieved perfect classification performance regardless of the number of variables. In contrast, the exploratory SP vs SN model showed high sensitivity but moderate specificity (Recall = 1.00, Specificity = 0.78, AUC = 0.94) (Table S4). Reduction to 5 proteins (PIGR, ACAN, PON3, ACM1, CFHR5) resolved this limitation.

Overall, these results indicate that serological stratification reveals more robust EV proteomic differences than analyses performed on RA patients as a single group. In addition, variable reduction improved model parsimony without compromising classification performance. Differences in disease activity did not explain the observed protein alterations.

### 3.5 Potential biological significance of EV protein profile in patients with SP RA

To explore the relationship between proteins enriched in patients with SP RA and clinical or immunological variables, correlation analyses were performed. Among the top 20 proteins identified in the SP versus HD comparison, all proteins showed significant correlations with at least one disease-specific autoantibody (Fig. S3C). In contrast, such associations were largely absent among the top 20 proteins identified in the SN-SP comparison (Fig. S3D).

**Fig. 4:**
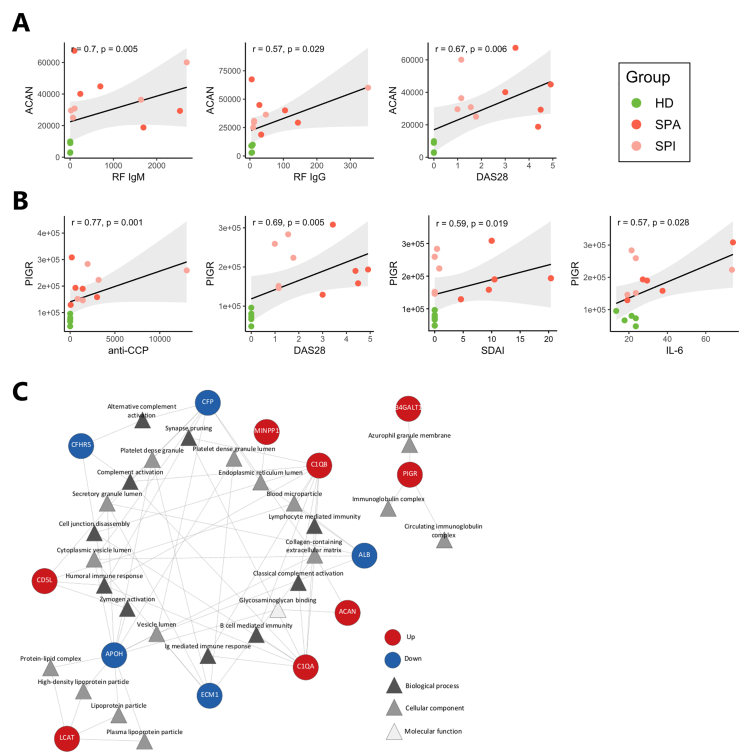
Biological associations and pathways of DEG proteins in patients with SP RA. **A-B.** Scatter plots illustrate significant correlations between the proteins ACAN (A) and PIGR (B) and the autoantibody titers, disease activity scores, and cytokine levels. Spearman's Rho correlation. The exact r and p values are shown in each graph. **C.** Gene–function interaction network integrating PLS-DA loading genes with significantly enriched Gene Ontology (GO) terms (padj <0.01). Gene nodes are colored by differential expression (red, positive fold change; blue, negative fold change), and GO terms are shown in shades of gray by ontology category (biological process, cellular compartment, and molecular function). Edges indicate gene–GO term associations from enrichment analysis.

Among the proteins with the highest discriminatory power, ACAN and PIGR exhibited significant positive correlations with relevant disease markers. ACAN was positively associated with RF IgM (r = 0.7, p = 0.005), RF IgG (r = 0.57, p = 0.029), and DAS28 (r = 0.57, p = 0.006), whereas PIGR correlated with IL-6 levels (r = 0.57, p = 0.028) (Fig. 4A–B). Although several cytokines were significantly increased in patients with SP RA (Table 1), correlations between cytokine concentrations and EV protein abundance were generally limited. Notable exceptions included PIGR and IL-6, as well as IGFBP3, which correlated with both IL-12p70 (r = 0.70, p = 0.0034) and TNF-α (r = 0.57, p = 0.0282) (Fig. S3C).

To further assess the clinical relevance of these proteins, Firth's penalized logistic regression, adjusted for age, was used to evaluate their associations with bone erosion and smoking status. No significant associations were identified for any of the proteins analyzed. However, CFHR3 (OR = 3.61; 95% CI = 0.78–26.41; p = 0.11) and IGFBP3 (OR = 1.71; 95% CI = 0.83–4.54; p = 0.15) showed a tendency toward increased abundance in smokers (Fig. S3E).

Functional network analysis of the top 20 proteins in the HD versus SP patients comparison highlighted the organization of EV-associated proteins into interconnected immune- and vesicle-related pathways, including complement activation, humoral immune response, and vesicle- and lipoprotein-associated compartments (Fig. 4C). Within this network, PIGR was primarily connected to proteins associated with immunoglobulin-containing complexes, supporting its role in the transport and transcytosis of antibodies and immune complexes (ICs), and suggesting a potential contribution to EV-mediated systemic distribution of immune cargo.

ACAN displayed broader connectivity, including links with complement-related components such as C1QA and C1QB, as well as proteins involved in extracellular matrix organization and glycosaminoglycan-binding pathways. Several proteins also converged on vesicle lumen and lipoprotein-related compartments, indicating that the observed protein signature is organized into coordinated EV-associated functional modules rather than isolated protein changes.

## 4 Discussion

This study provides a proteomic characterization of plasma-derived EVs in RA, stratified by serological status and disease activity, and compared with HD. While previous studies have focused on synovial fluid-derived EVs, plasma is a more accessible source with potential relevance for clinical translation. We observed differences in total EV protein content between patients and HD, particularly in SP individuals, consistent with the persistent proinflammatory profile described in this subgroup and potentially independent of DAS28-defined activity. LC-MS/MS analysis and PLS-DA cross-validation identified the specific enrichment of ACAN and PIGR in EVs from patients with SP RA.

Compared with HD, most EV proteins were decreased in patients. This global reduction may reflect systemic alterations in protein synthesis, secretion, or turnover associated with hepatic, metabolic, or cellular dysfunction in RA [35], although this interpretation remains speculative. While decreased albumin is the most evaluated feature in chronic conditions, other proteins may also be affected, particularly in patients with SP RA, where increased pro-inflammatory cytokines such as TNF-α could promote protein catabolism not only in muscle (cachexia) but also at the hepatic level [36].

ACAN has been previously described as an autoantigen in RA, whereas PIGR is involved in mucosal immunoglobulin transport. Their concurrent enrichment in EVs suggests a potential link between cartilage-derived antigens and mucosal immunity. ACAN is a major structural component of the cartilage extracellular matrix [37], and experimental models support its role as an autoantigen. Proteoglycan-induced arthritis in BALB/c mice leads to ACPA production and a Th1-skewed response with high IFN-γ levels [38]. In humans, T cell responses against citrullinated aggrecan correlate with autoantibody levels [39]. In our dataset, ACAN abundance in EVs increased (>2-fold) in RA and was positively correlated with autoantibodies. Although citrullinated ACAN peptides were not among the top modified proteins, these associations support their relevance as potential EV-derived autoantigens.

Current evidence supports the concept that RA may initiate at mucosal sites, where post-translational modifications such as citrullination trigger ACPA production and subsequent systemic autoimmunity [40]. PIGR mediates the transcytosis of polymeric IgA and IgM across mucosal epithelia [41]. Its extracellular cleavage product, the secretory component (SC), is elevated in early RA and correlates with ACPA levels and smoking status [42]. Increased PIGR expression has also been associated with periodontitis and viral infections such as Epstein–Barr virus, both linked to RA risk [[43], [44], [45]]. Inflammatory cytokines, including IL-1β, IL-17, IFN-γ, and TNF-α induce PIGR expression [46], consistent with our observation of positive associations with anti-CCP, IL-6, and DAS28. Experimental models further suggest that PIGR-mediated IgA transport can amplify inflammatory responses [47].

Although PIGR has not been previously reported in EVs from RA patients, it has been detected in EVs from cancer patients [48]. Its presence in EVs from patients with SP RA may reflect increased mucosal immune activation and suggest a potential mechanism for systemic dissemination of immunoglobulins or ICs. However, this hypothesis remains to be experimentally validated.

The integration of protein interaction networks and correlation analyses supports a coordinated organization of EV cargo around immune- and extracellular matrix-related pathways. These analysis indicate that EV-associated proteins are functionally structured rather than randomly distributed in RA. Within this framework, ACAN and PIGR occupy positions that link matrix remodeling to immune-related processes. Correlation analyses further support this observation, as several EV proteins were associated with autoantibodies (RF and anti-CCP), inflammatory mediators (CRP and IL-6), and disease activity (DAS28). Although these findings do not establish causal relationships, they suggest that circulating EVs may provide a snapshot of interconnected pathogenic processes relevant to RA. Importantly, the detection of autoantigens or citrullinated peptides in EVs should not be interpreted as evidence of disease development, since such molecules can also be detected in healthy individuals.

The findings of this work should be considered exploratory, as they require confirmation in independent, larger cohorts. The sample size of the proteomic analyses may have limited the ability to detect additional differences between groups. Furthermore, although PLS-DA and variable selection approaches enabled discrimination between clinical subgroups and facilitated the identification of candidate proteins, the supervised nature of these methods warrants cautious interpretation. Therefore, validating these results with complementary analytical approaches will be important for confirming the robustness of the proposed protein signatures.

Another aspect that merits further investigation is the characterization of other post-translational modifications (PTMs) in EV-associated proteins, since key RA-related autoantibodies recognize these modifications [49], including carbamylation and glycosylation. Future PTM-focused mass spectrometry of EVs may provide additional insights into their contribution to SP pathology.

## 5 Conclusions

The proteomic composition of plasma-derived EVs differs between patients with RA and HD, with serological status emerging as the principal source of biological variation. EV-associated ACAN and PIGR were consistently enriched in patients with SP RA and were associated with autoantibodies, inflammatory mediators, and proteins involved in extracellular matrix remodeling, but at a lower proportion with cytokines, suggesting that circulating EVs capture molecular signatures linked to both immune dysregulation and tissue damage.

Our findings support the concept that SP and SN RA represent biologically distinct disease subsets and highlight the potential value of EV proteomics for molecular patient stratification. While the present study was not designed to evaluate early diagnosis, prognosis, or treatment response, the identified protein signatures serve as candidate biomarkers warranting further investigation in longitudinal cohorts, including individuals at risk of developing RA and treatment-naïve patients.

Beyond their biomarker potential, the identified proteins may offer insights into pathogenic pathways connecting mucosal immunity, autoantibody generation, and joint tissue remodeling. Future studies integrating larger cohorts, functional validation, and characterization of post-translational modifications will be essential for clarifying the biological significance of these proteins and their roles in disease initiation and progression.

Overall, our results provide proof of concept that plasma EV proteomics can reveal clinically relevant molecular differences between RA subgroups. By identifying EV-associated proteins linked to seropositivity, this work lays the foundation for future translational studies to improve patient stratification, biomarker discovery, and the development of more personalized approaches to RA management.

## Declaration of generative AI and AI-assisted technologies

During the preparation of this manuscript, the authors used Grammarly and ChatGPT for grammatical review.

## CRediT authorship contribution statement

**Susana Castaño-López:** Formal analysis, Investigation, Methodology, Visualization, Writing – original draft. **Tulio J. Lopera:** Formal analysis, Investigation, Methodology, Visualization, Writing – original draft. **Valentina Restrepo:** Investigation, Methodology, Writing – review & editing. **Luisa F. Carbal:** Investigation, Methodology, Writing – review & editing. **Julieta M. Ramírez-Mejía:** Data curation, Formal analysis, Investigation, Methodology, Writing – review & editing. **Andrés Hernández:** Investigation, Methodology, Writing – review & editing. **Diana Gil:** Investigation, Methodology, Writing – review & editing. **Juan-Camilo Díaz:** Investigation, Methodology, Writing – review & editing. **Marcela Manrique-Moreno:** Formal analysis, Investigation, Methodology, Visualization, Writing – review & editing. **Lara Barazzuol:** Investigation, Methodology, Writing – review & editing. **Rafael Posada-Duque:** Investigation, Methodology, Writing – review & editing. **Mauricio Rojas:** Investigation, Methodology, Writing – review & editing. **Gloria Vásquez:** Investigation, Methodology, Writing – review & editing. **Lilu Corrales-Garcia:** Formal analysis, Investigation, Visualization, Writing – review & editing. **Julián D. Arias-Londoño:** Formal analysis, Investigation, Methodology, Validation, Visualization, Writing – review & editing. **Justina C. Wolters:** Data curation, Formal analysis, Investigation, Methodology, Visualization, Writing – review & editing. **Diana Castaño:** Conceptualization, Funding acquisition, Investigation, Methodology, Project administration, Supervision, Writing – original draft, Writing – review & editing.

## Declaration of competing interest

The authors declare the following financial interests/personal relationships which may be considered as potential competing interests: Diana Castaño reports that financial support was provided by the International Centre for Genetic, Engineering and Biotechnology (ICGEB). Diana Castaño reports financial support was provided by Minciencias, Colombian Government. If there are other authors, they declare that they have no known competing financial interests or personal relationships that could have appeared to influence the work reported in this paper.

## Data Availability

Data will be made available on request.
