# Supplementary material for: Aggrecan and polymeric immunoglobulin receptor in extracellular vesicles of patients with seropositive rheumatoid arthritis
Source: J Transl Autoimmun. 2026 Jul 8;13:100387. doi: 10.1016/j.jtauto.2026.100387 (PMC13400858; doi:10.1016/j.jtauto.2026.100387)
Supplement: Multimedia component 1 [file mmc1.pdf]

Supplementary figures

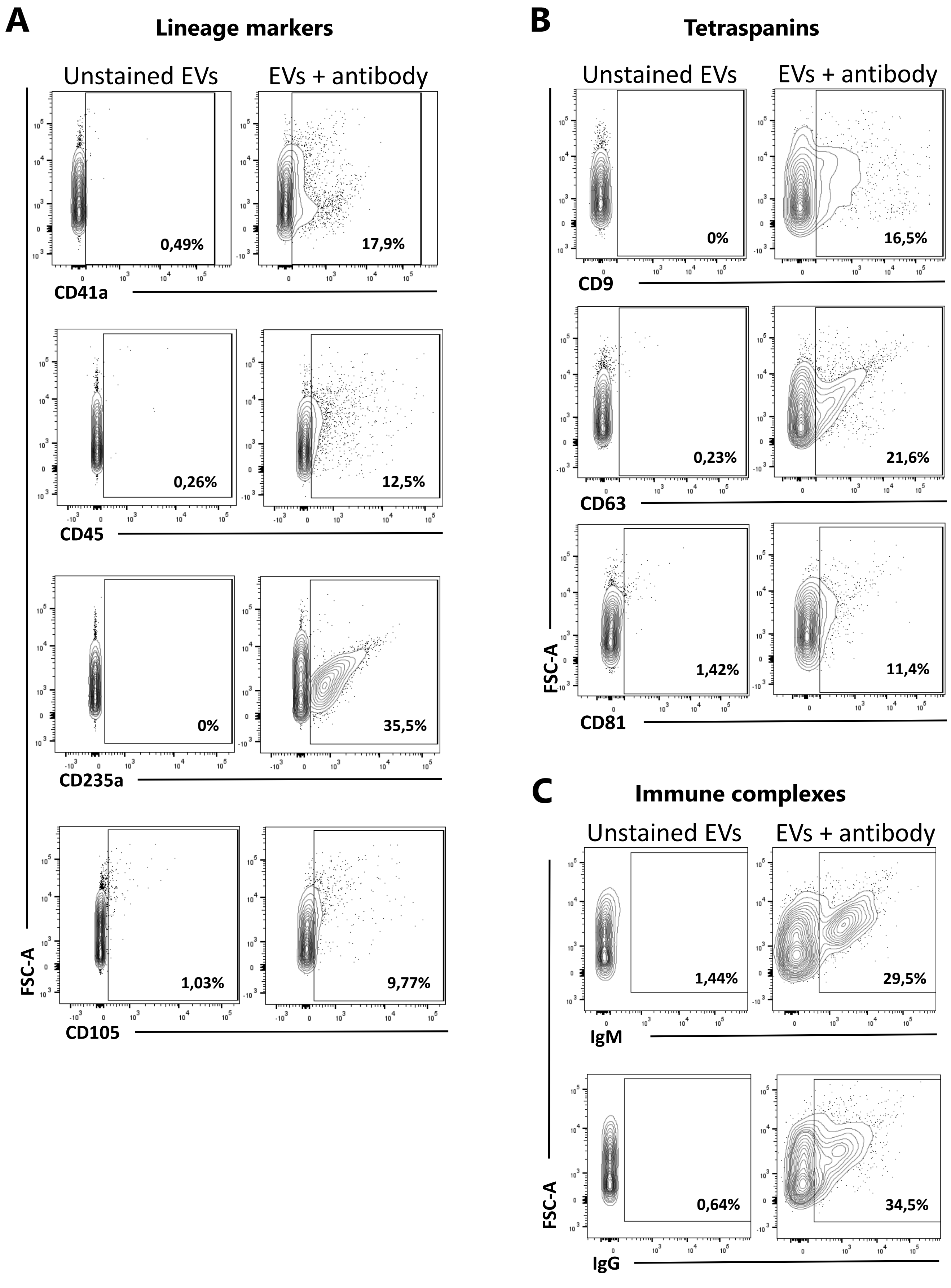

Figure S1. Gating strategy for analyzing EVs by flow cytometry. The frequencies of positive EVs for each marker were obtained by comparing EVs from the same individual with and without staining with specific antibodies against **A**) CD41a, CD235a, CD105, and CD45 (lineage); **B)** CD9, CD63 and CD81 (tetraspanins); and **C)** Constant fraction of IgM and IgG antibodies (immune complexes)

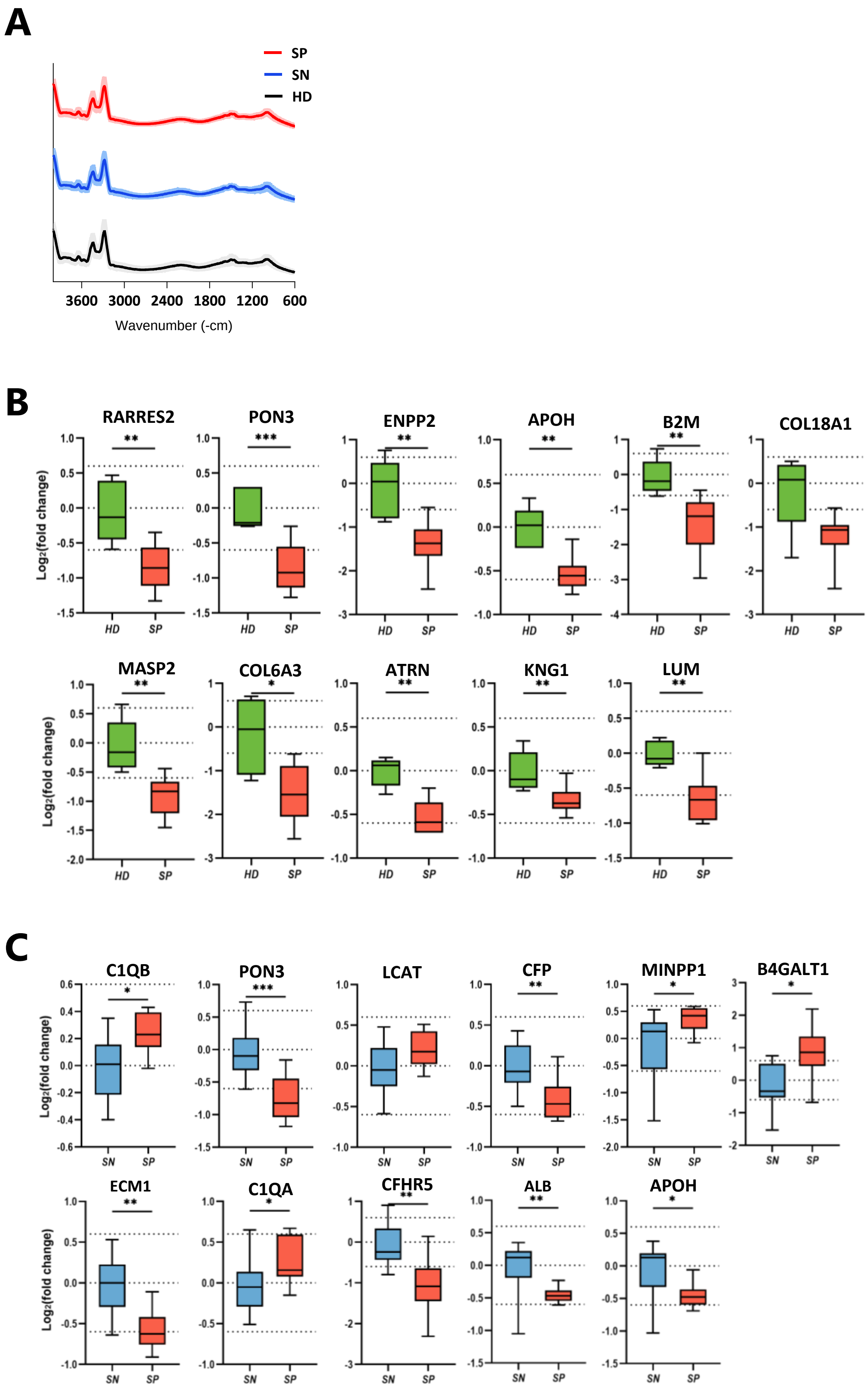

Figure S2. A. Fourier-transform infrared (FT-IR) spectra of EVs isolated from seropositive (SP), n = 10; seronegative (SN), n = 7; and healthy donor (HD), n = 5. Spectra are shown in the range of 4000–600 cm^{-1}. Shaded areas represent variability across samples within each group. **B.** Box plots and whiskers of fold change (FC) for top 20-sMC proteins between RA SP and HD, that had FC < 0.6. Dotted lines at ± 0.6 and zero. Mann-Whitney, *p ≤0.05; **p ≤0.01; ***p ≤0.001. **C.** Box plots and whiskers of fold change (FC) for top 20-sMC proteins between RA SP and RA SN, that had FC < 0.6. Dotted lines at ± 0.6 and zero. Mann-Whitney, *p ≤0.05; **p ≤0.01; ***p ≤0.001

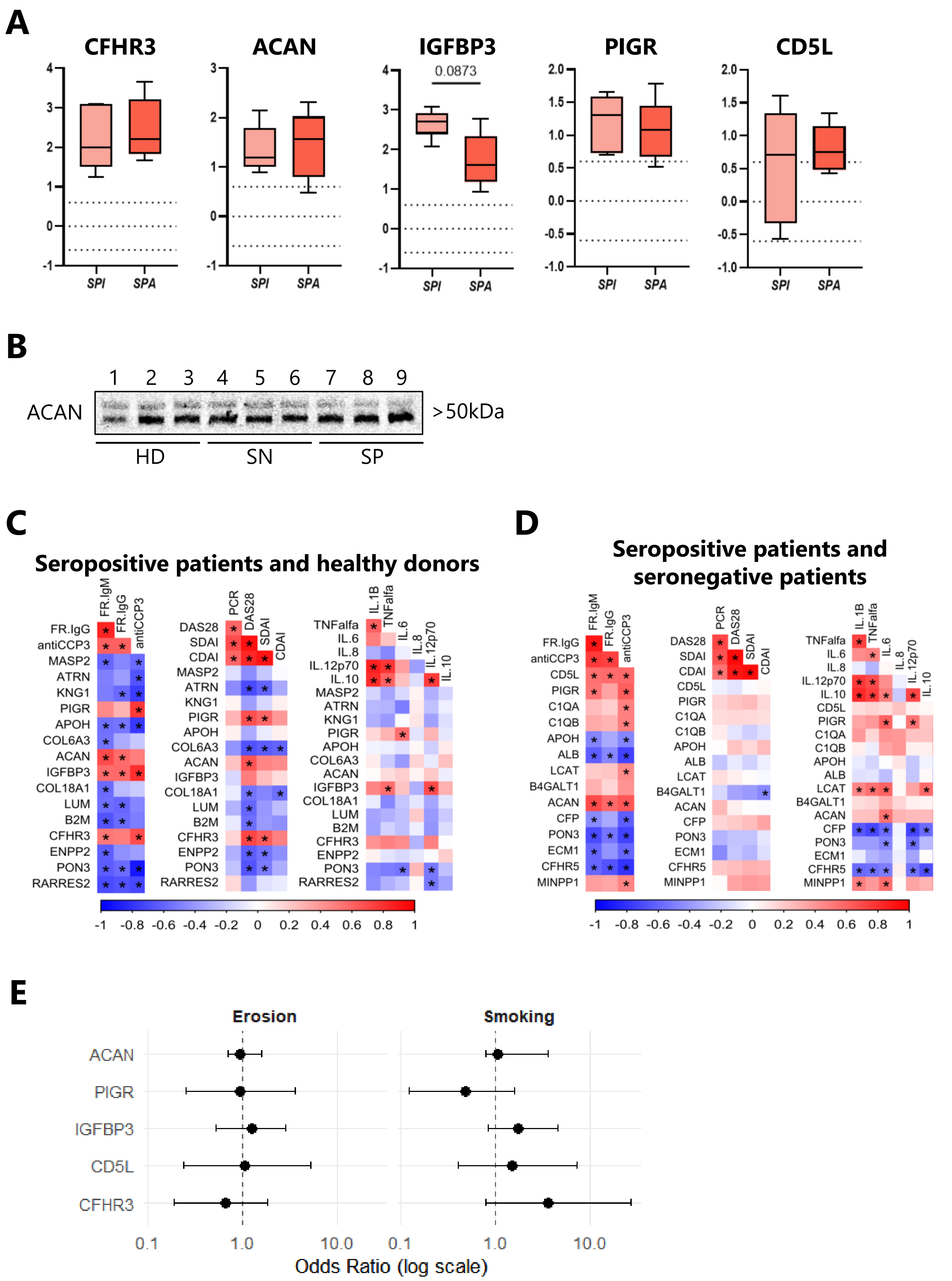

Figure S3. A. Box plots and whiskers of fold change (FC) for top 20-sMC proteins between RA SP and HD that had FC > 0.6 but separating RA SP according to the disease activity status (inactive disease, SPI, and active disease, SPA). Dotted lines at ± 0.6 and zero. Mann-Whitney comparison. **B.** Western blot showing ACAN levels in EVs from HD (n=3, lands 1, 2 and 3), SN RA patients (n=3, lands 4, 5 and 6), and SP RA patients (n=3, lands 7, 8 and 9). **C-D.** Correlation graphs between the 20 discriminating EV proteins (without immunoglobulins) and: autoantibody titters (left); RA activity markers (middle); and cytokine levels (right); in the comparison of patients with SP RA and HD (C) and patients with SN RA and SP RA (D). Spearman's Rho correlation. *p ≤0.05. Colors represent the r value of the correlation, where blue indicates negative correlation, and red indicates positive correlation. **E.** Forest plots showing odds ratios (95% CI) for the association between log2-transformed protein abundance and bone erosion (left) or smoking status (right), estimated using Firth's penalized logistic regression adjusted for age. Results should be interpreted as exploratory due to the small sample size.
